# Supplementary material for: The effects of genital myiasis on the diversity of the vaginal microbiota in female Bactrian camels
Source: BMC Vet Res. 2022 Mar 5;18:87. doi: 10.1186/s12917-022-03189-5 (PMC8897907; doi:10.1186/s12917-022-03189-5)
Supplement: Supplementary file 5 — Additional file 5. [file 12917_2022_3189_MOESM5_ESM.zip › MPL201709200_16s_yy/Treat1/css/js_css/jsPDF-master.zip!/jsPDF-master/examples/html2pdf/auto_break.html]

context2d auto-break


# Autobreak

The context2d plugin was tweaked to auto-break a document into multiple pages.

The auto-page-break feature allows vertical positioning by addressing the Y coordinate beyond the PDF page.

Note: *There is a margin bug that causes an item to not draw in the break.*

1. OL Item
2. OL Item
3. OL Item
4. OL Item
5. OL Item
6. OL Item
7. OL Item
8. OL Item
9. OL Item
10. OL Item
11. OL Item
12. OL Item
13. OL Item
14. OL Item
15. OL Item
16. OL Item
17. OL Item
18. OL Item
19. OL Item
20. OL Item
21. OL Item
22. OL Item
23. OL Item
24. OL Item
25. OL Item
26. OL Item
27. OL Item
28. OL Item
29. OL Item
30. OL Item
31. OL Item
32. OL Item
33. OL Item
34. OL Item
35. OL Item
36. OL Item
37. OL Item
38. OL Item
39. OL Item
40. OL Item
41. OL Item
42. OL Item
43. OL Item
44. OL Item
45. OL Item
46. OL Item
47. OL Item
48. OL Item
49. OL Item
50. OL Item
51. OL Item
52. OL Item
53. OL Item
54. OL Item
55. OL Item
56. OL Item
57. OL Item
58. OL Item
59. OL Item
60. OL Item
61. OL Item
62. OL Item
63. OL Item
64. OL Item
65. OL Item
66. OL Item
67. OL Item
68. OL Item
69. OL Item
70. OL Item
71. OL Item
72. OL Item
73. OL Item
74. OL Item
75. OL Item
76. OL Item
77. OL Item
78. OL Item
79. OL Item
80. OL Item
81. OL Item
82. OL Item
83. OL Item
84. OL Item
85. OL Item
86. OL Item
87. OL Item
88. OL Item
89. OL Item
90. OL Item
91. OL Item
92. OL Item
93. OL Item
94. OL Item
95. OL Item
96. OL Item
97. OL Item
98. OL Item
99. OL Item
100. OL Item
101. OL Item
102. OL Item
103. OL Item
104. OL Item
105. OL Item
106. OL Item
107. OL Item
108. OL Item
109. OL Item
110. OL Item
111. OL Item
112. OL Item
113. OL Item
114. OL Item
115. OL Item
116. OL Item
117. OL Item
118. OL Item
119. OL Item
120. OL Item
121. OL Item
122. OL Item
123. OL Item
124. OL Item
125. OL Item
126. OL Item
